# Supplementary material for: Persistence of Anti-SARS-CoV-2 Antibodies in Non-Hospitalized COVID-19 Convalescent Health Care Workers
Source: J Clin Med. 2020 Oct 1;9(10):3188. doi: 10.3390/jcm9103188 (PMC7600936; doi:10.3390/jcm9103188)
Supplement: Supplementary file 1 [file jcm-09-03188-s001.pdf]

Supplementary material

Supplementary tables

1) Supplementary Table 1: Patients' clinical characteristics

Clinical parameter	**COVID-19^{+}** Hospitalised ICU n=24	**COVID-19^{+}** Not-hospitalised n=16	**COVID-19^{-}** n=58
Male, n (%)	18 (75)	9 (56,2)	34 (58,6)
Age, mean (± SD)	64 (±8)	53 (±9)	53 (±9)
Comorbidities, n (%)	11 (45,8)	2 (12,5)	-
Obesity	3 (27,2)	1 (6,2)	-
Cardiopulmonary	7 (63,6)	2(12,5)	-
Diabetes	5 (45,4)	-	-
Renal	-	-	-
Vasculopathy	3 (27,2)	1 (6,2)	-
Multiple	7 (63,6)	1 (6,2)	-

2) Supplementary Table 2: COVID+ non-hospitalized patients clinical symptoms

	sex	disease duration (d)	fever	ageusia	anosmia	fatigue	mialgia	artalgia	dhiarrea	coryza	cough	dyspnea
1	M	14	y	y	y	y	y	y	y	n	y	n
2	M	21	y	y	y	y	y	n	n	n	y	y
3	M	14	y	y	y	y	y	n	n	y	n	n
4	M	30	y	y	y	y	n	n	n	y	y	n
5	M	21	y	y	y	y	y	n	n	y	y	n
6	M	28	y	y	y	y	y	y	n	y	y	n
7	M	35	y	y	y	y	y	n	y	y	y	n
8	F	14	y	y	y	y	y	y	n	y	y	n
9	F	16	y	y	y	y	y	n	n	n	y	n
10	F	21	y	y	y	y	y	y	y	n	y	y
11	F	21	y	y	y	y	y	n	n	n	y	n
12	F	16	y	y	y	y	y	n	y	n	y	n
13	F	28	y	y	y	y	y	n	y	n	n	n
14	M	21	y	y	y	y	n	n	y	n	n	n
15	M	28	y	y	y	y	y	n	y	n	n	n
16	F	28	y	y	y	y	n	n	n	n	n	n

3) Supplementary Table 3: Luminex analytes.

	**Analyte**		**Analyte**
**Cytokines**	IL-1alpha	**Chemoattractors**	CXCL1 (GROa)
	IL-1beta		CXCL8 (IL-8)
	IL-1Ralpha		CXCL10 (IP-10)
	IL-2		CXCL12 (SDF-1)
	IL-4		CCL2 (MCP-1)
	IL-5		CCL3 (MIP1a)
	IL-6		CCL4 (MIP-1b)
	IL-7		CCL5 (Rantes)
	IL-9		CCL11 (Eotaxin)

	IL-10		
	IL-12p70		
	IL-13		
	IL-15	**Growth factors**	LIF
	IL-17A		BDNF
	IL-18		NGFbeta
	IL-21		GM-CSF
	IL-22		HGF
	IL-23		EGF
	IL-27		PDGFBB
	IL-31		SCF
	IFN-alpha		FGF-2
	IFN-gamma		PLGF
	TNF-alpha		VEGF-A
	TNFbeta/LTA		VEGF-D

Supplementary figures

A

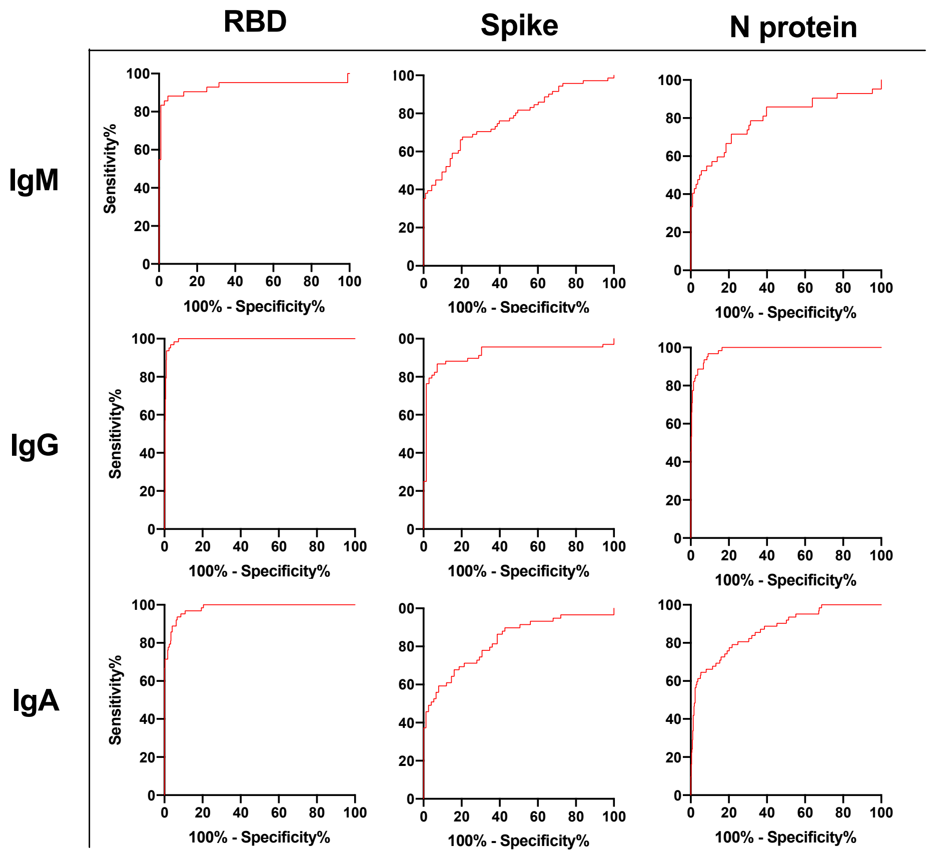

B

		Threshold (OD)	Sensitivity (%)	Specificity (%)
**RBD**	IgM	0.186	91	87
	IgG	0.277	95.2	97.64
	IgA	0.295	95.2	91.5
**Spike**	IgM	0.277	76	56
	IgG	0.3	77	98.55
	IgA	0.1914	71	73
**N**	IgM	0.2461	81	62
	IgG	0.326	95	91
	IgA	0.381	69	85

<u>*Supplementary Figure 1: ROC curves*</u> (A) ROC curves determining specificity and sensitivity for the different assays (B) summary table of the OD threshold values and corresponding specificity and sensitivity.

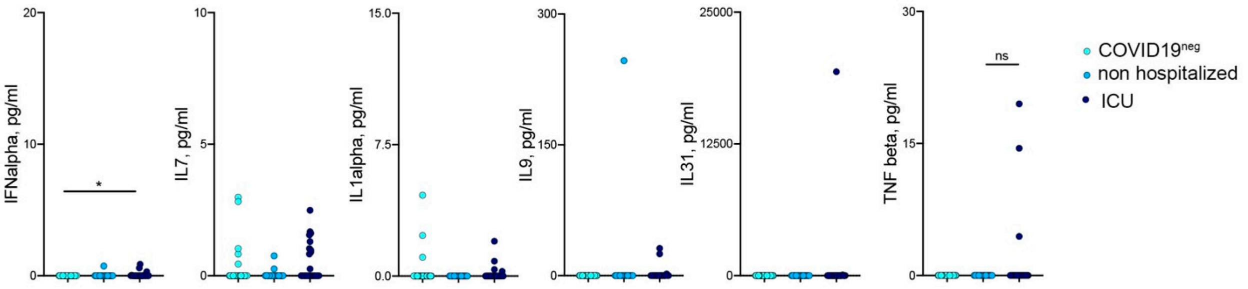

Supplementary Figure 2: Cytokine levels in sera of COVID19+ patients. Cytokines not significantly different between hospitalized (dark blue) and non- hospitalized (blue) COVID+ patients. Statistical significance was calculated using Kruskal-Wallis nonparametric test for multiple comparisons. P < 0.05 (*) were regarded as statistically significant.

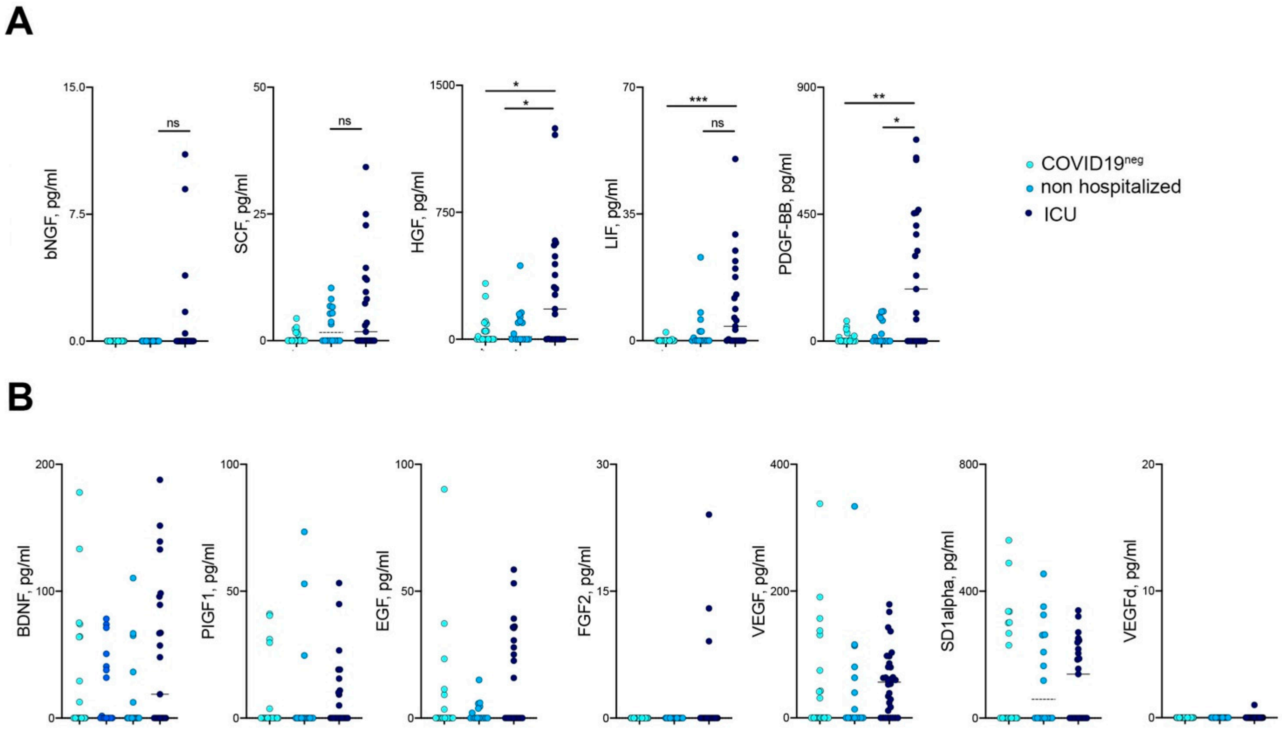

Supplementary Figure 3: (A) Growth factors present in ICU hospitalized (dark blue) but not in non-hospitalized (blue) COVID+ patients (B) Growth factors not significantly different between ICU hospitalized (dark blue) and non-hospitalized (blue) COVID+ patients. Statistical significance was calculated using Kruskal-Wallis nonparametric test for multiple comparisons. P < 0.05 (*), P < 0.01 (**) were regarded as statistically significant.

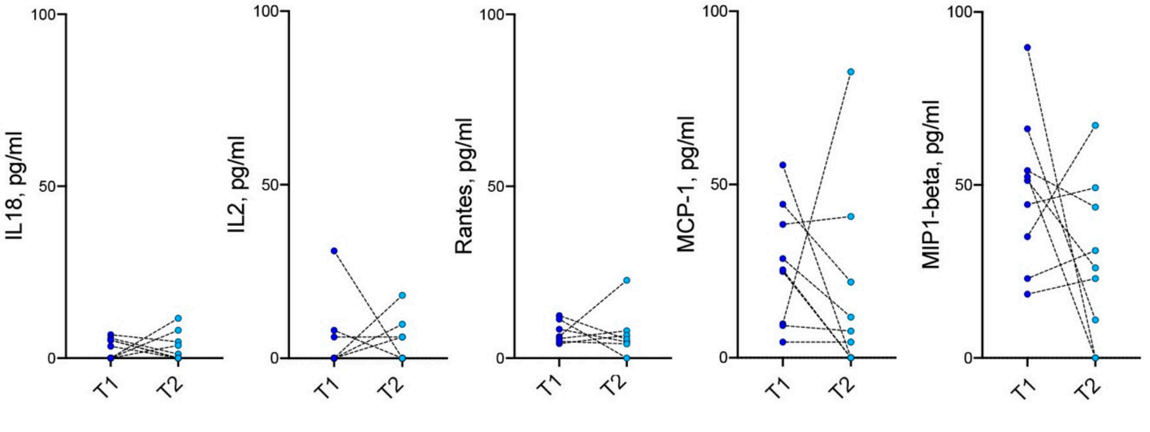

Supplementary Figure 4: Not significant longitudinal variation of serum cytokines and chemokines in non-hospitalized COVID 19+ patients.
